# Differences in Characteristics of Error-Related Potentials Between Individuals With Spinal Cord Injury and Age- and Sex-Matched Able-Bodied Controls

**DOI:** 10.3389/fneur.2018.01192

**Published:** 2019-01-31

**Authors:** Philipp Keyl, Matthias Schneiders, Christian Schuld, Steffen Franz, Maximilian Hommelsen, Nobert Weidner, Rüdiger Rupp

**Affiliations:** Spinal Cord Injury Center, Heidelberg University Hospital, Heidelberg, Germany

**Keywords:** neurophysiology, evoked potentials, electroencephalogram, error potentials, spinal cord injury, brain-computer interface

## Abstract

**Background:** Non-invasive brain-computer interfaces (BCI) represent an emerging technology for enabling persons with impaired or lost grasping and reaching functions due to high spinal cord injury (SCI) to control assistive devices. A major drawback of BCIs is a high rate of false classifications. The robustness and performance of BCIs might be improved using cerebral electrophysiological correlates of error recognition (error-related potentials, ErrPs). As ErrPs have never been systematically examined in subjects with SCI, this study compares the characteristics of ErrPs in individuals with SCI with those of able-bodied control subjects.

**Methods:** ErrPs at FCz and Cz were analyzed in 11 subjects with SCI (9 male, median age 28 y) and in 11 sex- and age-matched controls. Moving a shoulder joystick according to a visual cue, subjects received feedback about the match/mismatch of the performed movement. ErrPs occurring after “error”-feedback were evaluated by comparing means of voltage values within three consecutive time windows after feedback (wP1, wN1, wP2 containing peak voltages P1, N1, P2) using repeated-measurement analysis of variance.

**Results:** In the control group, mean voltage values for the “error” and “correct” feedback condition differed significantly around N1 (FCz: 254 ms, Cz: 252 ms) and P2 (FCz: 347 ms, Cz: 345 ms), but not around P1 (FCz: 181 ms, Cz: 179 ms). ErrPs of the control and the SCI group showed similar morphology, however mean amplitudes of ErrPs were significantly smaller in individuals with SCI compared to controls for wN1 (FCz: control = −1.55 μV, SCI = −0.27 μV, *p* = 0.02; Cz: control = −1.03 μV, SCI = 0.11 μV, *p* = 0.04) and wP2 (FCz: control = 2.79 μV, SCI = 1.29 μV, *p* = 0.011; Cz: control = 2.12 μV, SCI = 0.81 μV, *p* = 0.003). Mean voltage values in wP1, wN1, and wP2 did not correlate significantly with either chronicity after or level of injury.

**Conclusion:** The morphology of ErrPs in subjects with and without SCI is comparable, however, with reduced mean amplitude in wN1 and wP2 in the SCI group. Further studies should evaluate whether ErrP-classification can be used for online correction of false BCI-commands in individuals with SCI.

## Introduction

A spinal cord injury (SCI) and the associated impairment of motor functions below the level of injury represents an unexpected and life-altering condition leading to limited autonomy and participation in professional and private life of an affected person. In particular, a lesion of the high cervical spinal cord with restrictions of upper extremity functions up to complete tetraplegia is experienced as highly disabling and results in high dependency on caregivers (1, 2). If severe impairments of hand function persist in the chronic stage after SCI, the loss of motor functions is substituted or compensated by the use of assistive devices (ADs). For successful operation of electronic ADs such as computers, upper extremity neuroprostheses or robot arms, the human-machine interface plays a crucial role. Traditional user interfaces such as joysticks or keyboards rely on some preserved hand function, which might not be present in persons with very high SCI. Brain-computer interfaces (BCIs) are an emerging technology that hold the potential for enabling such end users to control ADs (3). Non-invasive BCIs based on the real-time analysis of the electroencephalogram (EEG) are most promising for everyday applications in end users due to their ease of use by caregivers, the broad availability of the hard- and software components and the low costs (4). Examples of BCIs applied in end users with SCI are (1) the synchronous P300-BCI for control of a virtual keyboard (5) or an electrical wheelchair (6), and (2) the asynchronous BCI based on modulations of sensorimotor rhythms induced by motor imagery for control of, e.g., a grasp neuroprosthesis using electrical stimulation to reactivate paralyzed muscles (7–9) or a robotic arm (10).

A major drawback of current non-invasive BCIs, which severely limits their translation from the lab into real world application at end users' homes, is the high rate of false positive commands in particular in end users with only moderate performance (11, 12). Most notably, false-positive decisions leading to an erroneous behavior of the controlled AD are a cause for frustration in end users feeling not being in control (3).

Numerous studies have identified electrophysiological correlates of error recognition in the human brain (error-related potentials, ErrPs) which can be successfully detected and used for error correction (13). Consequently, ErrPs detected on a single trial basis might contribute to overcome the current limitations of non-invasive BCIs.

The term ErrP has been established in the context of BCI-research and summarizes different EEG potentials which follow a mistaken action in various manifestations (14, 15). ErrPs are potentials that occur in the EEG when a person realizes an own mistake [“response ErrP” (16)], e.g., an erroneous movement, as well as when external feedback about a mistake in an action is received, e.g., by a signal indicating an erroneous movement [then also called “feedback ErrP” (16, 17)]. As an ErrP occurs even if the origin of the error lies within the operated device, e.g., a BCI (18), it [in this case specified as “interaction ErrP” (16)] may be used as part of a correction mechanism. If, for example, the user of a BCI-controlled neuroprosthesis has received feedback on a non-intended command before an undesired action of the neuroprosthesis is executed, the occurrence of an ErrP could correct or abort the generation of this unwanted movement. By this, the inclusion of ErrPs in a BCI-based control scheme consequently follows the concept of the hybrid-BCI (hBCI) combining a BCI with additional input signals (even from the same device) to improve robustness and control accuracy (19). Studies based on this approach of ErrP classification have shown to significantly improve the efficiency of traditional human-computer interfaces such as a button (17) or a thumbstick of a gamepad (20). Recent studies involving mainly able-bodied subjects have confirmed that also the performance of a motor imagery-based BCI for robotic arm (21, 22) or game control (23), and also a P300-speller (24) can be enhanced by the implementation of ErrP-detection. It can be expected that the BCI-performance also of subjects with SCI might be increased by an ErrP-based correcting mechanism.

The brain regions most often discussed as sources of error processing are the anterior cingulate cortex, anterior insula, inferior parietal lobe and intraparietal sulcus, but many other regions of the cortex, subcortex and cerebellum are also assumed to be involved in error processing (25). SCI results in an essential anatomical and functional reorganization of the central nervous system (CNS) including decrease in cortical gray matter volume, which affects in particular motor areas innervating the paralyzed body parts (26–28). Studies investigating these SCI-associated changes on brain activity observed an attenuation of amplitudes of evoked potentials (29, 30) and power density in the EEG frequency band of 8–13 Hz (31), which is known to be highly related to sensorimotor activity in the brain (32).

Since brain potentials have been shown to be altered in people with SCI, this might also be true for ErrPs potentially negatively affecting their correct classification. Systematic investigations on the characteristics of ErrPs in people with SCI represent the groundwork to facilitate this field of applied science, however, the available knowledge is sparse and mainly based on single case studies only. Therefore, the aim of this study is to shed light on the question, as to whether the morphology and amplitude of ErrPs in SCI are altered in individuals with SCI compared to able-bodied control subjects.

## Methods

### Participants

This prospective, exploratory matched-pair controlled study was carried out according to the Declaration of Helsinki. Its protocol was approved by the Ethical Committee of the University of Heidelberg (vote number: S-267/2015; registered in the German Clinical Trial Register (http://www.drks.de/) with ID DRKS00010290). All participants provided written informed consent according to the Declaration of Helsinki. Inclusion criteria for the group of subjects with SCI and the control group were both sexes and an age between 18 and 50. Subjects with SCI were included, if the SCI [American Spinal Injury Association (ASIA) impairment scale (AIS) grade A–D (33)] was persisting for at least 3 months post-injury and if they had preserved shoulder function (motor level at or below the spinal segment C4). Further diseases and medication were documented. Able-bodied participants were included in the control group if they were free from chronic diseases and did not receive permanent medication. Exclusion criteria for both groups were epileptic seizures or psychiatric diseases in their medical history.

Every person with SCI was matched to an able-bodied person of the same sex with a maximum age difference of 5 years.

### Experimental Setup

To induce ErrPs, a movement task was developed in which the participant operated a simple input device, namely a 2-axis shoulder joystick. We decided to use a shoulder joystick because it represents the standard input device of grasp neuroprostheses as well as a component of an hBCI in an arm neuroprosthesis (19). It allows end users with a motor level caudal to C3 to reliably select one of two movement directions (pro-/retraction—elevation/depression). The shoulder joystick was attached to the right shoulder. Participants of the control group were seated in a chair in front of a computer screen, while individuals with SCI were sitting in their wheelchairs. In a first step, the maximum shoulder range of motion in the two directions of interest was determined.

### Trials

**Figure 1:**
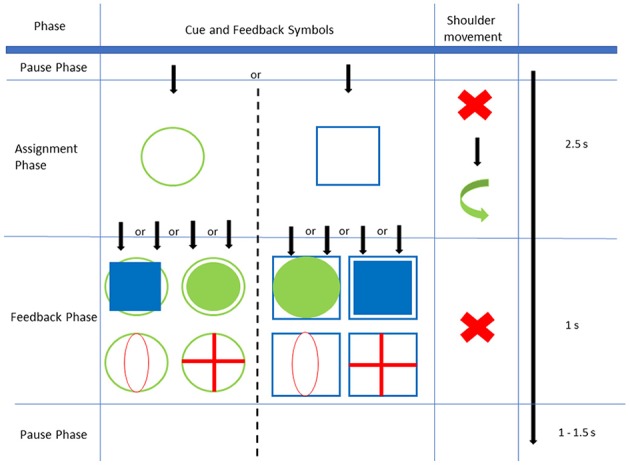
Experimental Setup: In the assignment phase, either an empty green circle or empty blue square is shown as a cue and must be followed by the correct shoulder movement within 2.5 s. During feedback phase one of four feedbacks is shown for 1 s. Afterwards, there is a pause phase of variable length.

The main experiment consisted of 6 runs (M1–M6) with 80 trials each. Each trial consisted of three phases: an assignment phase (2.5 s), a feedback phase (1 s), and a pause phase (1–1.5 s). Throughout the assignment phase, a visual cue was presented to the participant on the screen, indicating the direction the shoulder should be moved to, either upward (elevation, empty green circle) or forward (protraction, empty blue square). The performed movement had to exceed a certain threshold that had been set to 60% of the maximum range of motion. The joystick registered movements only while the visual cue was presented (i.e., in assignment phase). The participant was instructed to finish the movement (return to neutral position) within the assignment phase and to avoid any movement other than of the shoulder. Thus, any movement-related influence on the EEG is avoided during the subsequent feedback phase. In this phase the participant was informed about the direction and extent of the movement registered by the joystick by a symbol overlaying the initial visual cue (Figure 1):

- upward movement above the threshold → filled green circle
- forward movement above the threshold → filled blue square
- combined movement above the thresholds of both axes → red plus
- movement below the thresholds of both axis → red zero

Converse feedback (i.e., a filled green circle following the cue of a blue square or a filled blue square following the cue of a green circle), and the symbols “red zero” and “red plus” indicated a wrong movement and were expected to be followed by an ErrP in the EEG. Accordingly, the EEG signal that followed a feedback of a correct movement (indicating successful movement) was named “correct potential” (CorrP). At the end of the feedback phase, the screen was blanked for 1–1.5 s, afterwards a new trial was started with the visual cue of a green circle or a blue square.

### Runs

First we conducted three runs (E1–E3) during which the participants performed (E1) horizontal eye movements, (E2) vertical eye movements, and (E3) eye blinking. These runs were used later to remove EEG artifacts associated with eye movements as described in the next section.

Before the main experiment with its 6 runs (M1–M6), an additional run (visual feedback = VF) consisting of 80 trials was performed during which every combination of visual cues and feedback was shown 10 times with participants not knowing about the subsequent task. The aim of this run was to measure visually evoked potentials caused by the visual cues for later removal. Similar to the main experiment, the participants should not move while observing the visual feedback. After explanation of the tasks, the participants conducted 15 trials to confirm that they had understood the task.

In three runs (M1–M3) of the main experiment, the feedback appeared independently of the joystick input, i.e., the participants had no influence on the feedback during these runs. By this approach, systematic differences between participants in terms of the number of feedback about incorrect movements were compensated. Erroneous feedback appeared in 15% (M1), 20% (M2), and 25% (M3) during these runs. In the remaining 3 runs (M4–M6), the feedback was not adjusted by the computer and the participants received the true feedback about the correctness of their movements. All of these experimental conditions were chosen to investigate the impact of an erroneous user interface on the characteristics of the ErrPs. The participants were unaware of the differences between the runs. The order of all six runs of the main experiment (M1–M6) was randomized.

### EEG Analysis

Brain signals were acquired using a g.GAMMAcap with 64 active electrodes (g.tec medical engineering GmbH, Schiedlberg, Austria) arranged in the international 10–10 system covering the entire scalp. EEG was referenced against the ground electrode which was positioned on the forehead. Signals were recorded with a sampling rate of 512 Hz and amplified with a multichannel EEG-amplifier (g.HIamp, g.tec medical engineering GmbH, Schiedlberg, Austria). Impedances of the electrodes were kept below 30 kΩ and checked after each run. EEG data was down-sampled to 128 Hz and band-pass-filtered (4th order Butterworth) between 1 and 10 Hz.

In a first step, artifacts were removed manually by visual inspection of the recorded signals. In a second step, we applied an algorithm to remove artifacts caused by eye movements and eye blinks based on Parra et al. (34) using the data from runs E1, E2 and E3.

For evaluation of time series, we used the gaitcad toolbox (35) in MATLAB (The Mathworks, Natick, MA, USA). The EEG after start of the feedback phase was averaged for ErrP and CorrP conditions and for each electrode, run and person. Averaged visually evoked potentials for correct and error conditions (recorded during VF) were subtracted from every ErrP and CorrP condition for the whole feedback phase, respectively (36).

For the following reasons, we evaluated the ErrP and CorrP as separate components, rather than the difference of both, which was performed previously by other authors [e.g., (37)]: First, the CorrP is not a real baseline, but must be seen as the specific electrophysiological correlate when feedback about a correct action occurs (36). Therefore, computing the difference leads to information loss. Also, if ErrPs should be detected asynchronously in single trials, the difference between the ErrP and the baseline EEG is what matters for classification. Second, it is not known, how the ErrP or the CorrP may be changed in individuals in spinal cord injury. For instance, in patients with lateral prefrontal brain damage, there can be error processing even in correct trials (38). By evaluating both, erroneous and correct trials separately, changes can be ascribed to either one or both.

**Figure 2:**
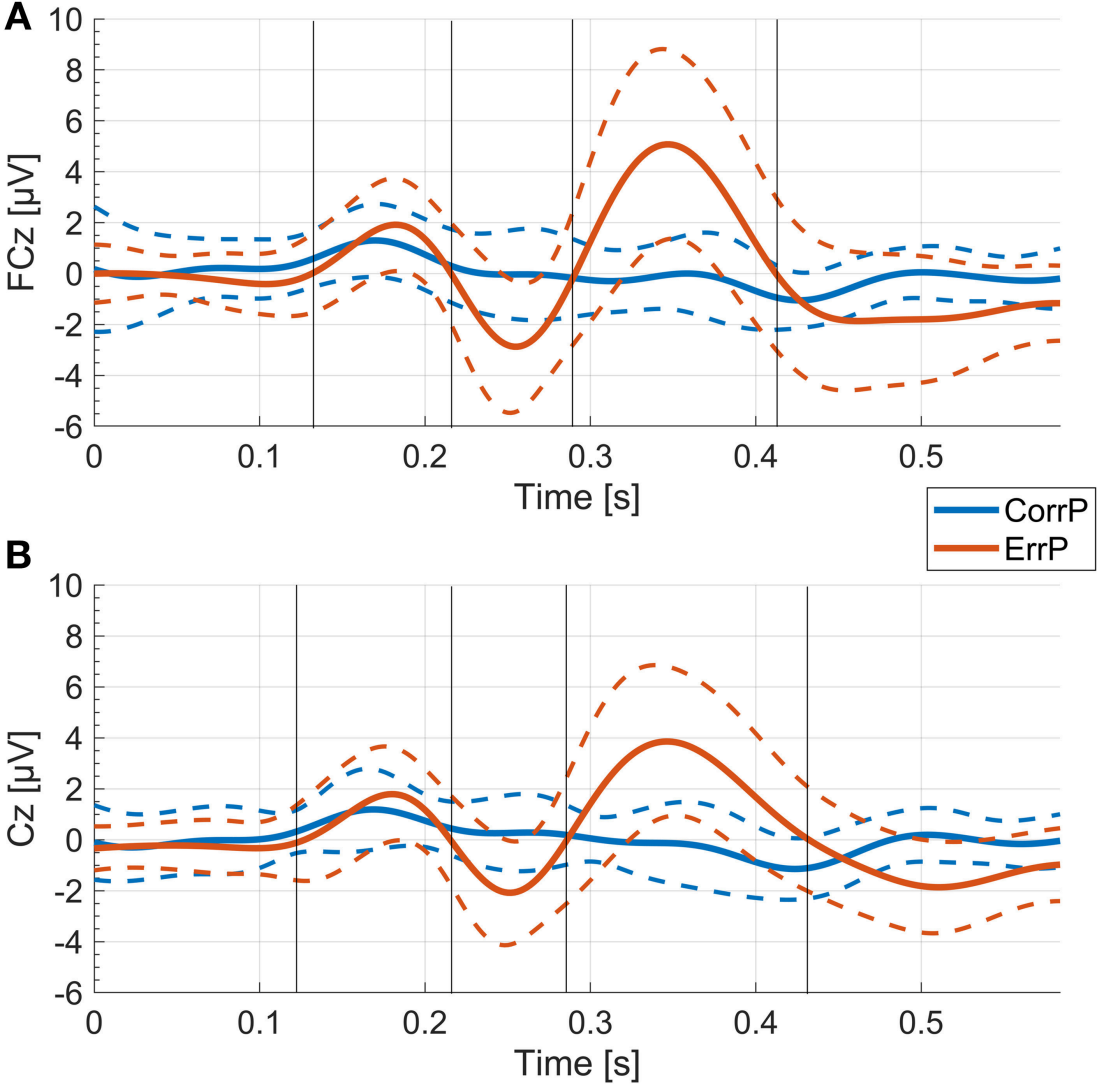
ErrP and CorrP of the control group. Gray vertical lines indicate the time windows that were used for calculation of the mean voltage values (wP1, wN1, wP2) for each individual. **(A)** Mean and standard deviation (dashed lines) of all participants in the control group of the averaged single trial EEG of each participant at FCz. **(B)** Mean and standard deviation (dashed lines) of all participants in the control group of the averaged single trial EEG of each participant at Cz.

We specifically examined electrodes FCz and Cz which are located above the origin of the ErrP over the anterior cingulate cortex (17). For evaluation of the different peaks, we analyzed the mean voltages in the time windows located around the time points of the peaks P1, N1, and P2. These time windows were defined by the zero-crossings of the mean ErrP of the control group (Figure 2), similar to previous descriptions in psychological studies investigating the effect of psychiatric disorders on error-related negativity (39–42). We preferred this approach over the peak-to-peak method (43, 44) which only analyzes differences between minimum and maximum peaks and does not allow for differentiation of changes of each of the peaks. Additionally, we considered the time window-based analysis to be less prone to artifacts or outliers than focusing on single peaks. To distinguish explicitly between the time windows around the peaks and the time points of the defining peaks, we refer to the time windows as wP1, wN1 and wP2, respectively.

### Statistical Analysis

Statistical analysis was performed using Statistica 7.1 (Statsoft, Tulsa, OK, USA), R statistical packages 3.4.0 (45) and Matlab 2017b Statistics Toolbox (The MathWorks, Natick, MA, USA). Single trials were first averaged for each participant, and run and then the averaged ErrP and CorrP were used for ANOVA, thus weighting the ErrP and CorrP of every individual equally. Mean voltage values of ErrP were analyzed using repeated-measurement analysis of variance (ANOVA) with the levels “group” (SCI or control), “feedback” (error or correct), and “repetition” (runs M1 to M6). Fisher's Least Significant Difference (LSD) was used as *post-hoc* test. We tested for differences between runs with and without influence with an additional ANOVA with levels “group” (SCI or control) and “influence” (influence on feedback result or no influence). Numbers of errors were compared between groups using the Mann-Whitney U-test. Pearson's correlations of mean voltage values of wP1, wN1, and wP2 with time after SCI onset and level of injury were analyzed. All tests were performed as two-sided tests with alpha = 0.05.

## Results

**Figure 3:**
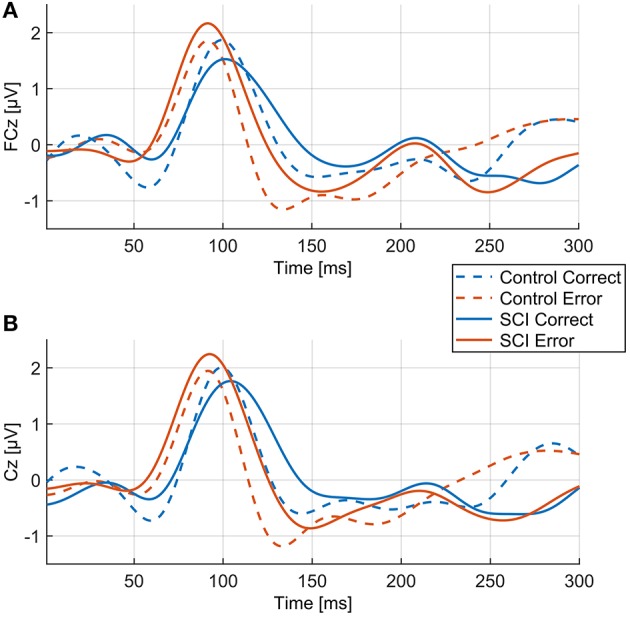
Mean EEG voltage values during visual feedback (VF) for “error” and “correct” feedback of the control and the SCI group. **(A)** FCz, **(B)** Cz.

In total, 13 subjects with SCI and 13 matched able-bodied controls participated in the study. Two participants with SCI had to be excluded from the study, as they felt unable to sit without pain for the whole time of the experiment. Eleven individuals with SCI (9 male, median age 28 y, range 19 to 48 y) completed the study and were matched to 11 control subjects (median age 24 y, range 22 to 49 y). The demographic and clinical data of participants with SCI are presented in Table 1. All participants asserted that they had felt to be in control of the joystick-feedback mechanism. The analysis of the EEG in the VF run prior to the main experiment revealed, that visual feedback results in one distinct peak that coincided with the P1 peak of the ErrP (Figures 2, 3).

**Table 1:** Characteristics of study participants in the SCI group.

**Patient**	**AIS**	**Neurological level of injury**	**Time since injury**	**Age range**	**Medication**
1	A	C4	>15 years	35–39	a, b, d, e
2	B	C6	3–6 months	20–24	a
3	A	Th6	6–12 months	25–29	c, d, e
4	A	Th7	6–12 months	20–25	a, e
5	A	C6	>15 years	45–49	–
6	D	L1	3–6 months	20–24	–
7	A	Th6	6–12 months	35–39	a, c, d, e
8	A	C4	>5 years	20–24	a
9	B	C5	3–6 months	18–19	a, b, c, e
10	A	Th10	3–6 months	45–49	a
11	A	C6	> 15 years	35-39	d, e

*CNS effective medication are categorized into “a” (opioids), “b” (benzodiazepines), “c” (anticonvulsants), “d” (spasmolytics), and “e” (anticholinergics). AIS, American Spinal Injury Association (ASIA) Impairment Scale. Ranges are given for time since injury and age to exclude any potential identification of subjects*.

In those runs, in which participants had influence on the feedback, the direction of movements of the shoulder joystick were more often incorrect in persons with SCI compared to control subjects, although the differences are not significant (control: median: 28.8%, IQR = 19.3%, range = 12.5%-83.3%, SCI: median: 47.9%, IQR = 31.3%, range = 13.8%-78.8%, p = 0.066, Mann–Whitney U-test).

**Figure 4:**
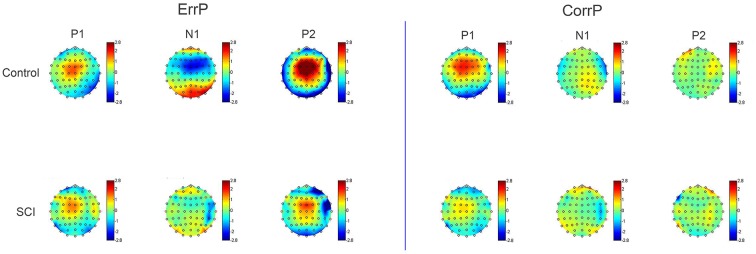
Topographic map of voltage distribution on the scalp at time points P1 (181 ms), N1 (254 ms), and P2 (347 ms). The peaks of the error potential are more distinctive and higher in amplitude in the control group than in the SCI group. CorrPs only show one positive peak at the same time point as the ErrP's P1.

Averaged over all control participants, the ErrP of the control group consisted of three peaks, each with a maximum over FCz (Figures 2, 4). At FCz, a small positive peak (P1, peak: 181 ms, zero-crossings: 132 and 214 ms), followed by a large negative peak (N1, peak: 254 ms, zero-crossings: 215 and 289 ms) and a large positive peak (P2, peak: 347 ms, zero-crossings: 290 and 412 ms) were detected. At Cz, similar to FCz, the control ErrP consisted of P1 (peak: 179 ms, zero-crossings: 127 and 215 ms), N1 (peak: 252 ms, zero-crossings: 216 and 285 ms) and P2 (peak: 345 ms, zero-crossings: 286 and 431 ms).

**Figure 5:**
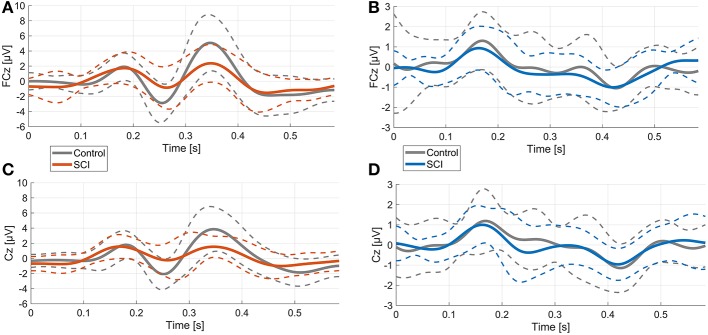
Comparison of ErrPs and CorrPs between control and SCI group. Thick lines represent mean voltage values of each group, thin dotted lines indicate standard deviation. **(A)**, **(B)** FCz; **(C)**, **(D)** Cz.

In the control group, the mean voltage amplitudes between ErrP and CorrP differed significantly for wN1 and wP2, but not wP1 (electrodes FCz and Cz, Figure 2, Table 2). In the SCI group, the mean amplitudes differed significantly between ErrP and CorrP for wP2, but not for wP1 and wN1 at FCz and Cz (Figure 5, Table 2).

**Table 2:** Mean voltage values for time windows wP1, wN1, and wP2 at electrodes FCz and Cz.

		**Control**			**SCI**			**ErrP**	**CorrP**
		**ErrP [μV]**	**CorrP [μV]**	***p***	**ErrP [μV]**	**CorrP [μV]**	***p***	**Control vs. SCI *p***	**Control vs. SCI *p***
FCz	wP1	1.1	0.91	0.65	1.20	0.67	0.22	0.81	0.59
	wN1	−1.55	−0.01	0.007*	−0.27	−0.11	0.76	0.02*	0.84
	wP2	2.79	−0.23	< 0.001*	1.29	−0.38	0.005*	0.011*	0.79
Cz	wP1	1.02	0.91	0.80	1.06	0.68	0.36	0.91	0.58
	wN1	−1.03	0.28	0.02*	0.11	−0.16	0.60	0.04*	0.41
	wP2	2.12	−0.34	< 0.001*	0.81	−0.26	0.02*	0.003*	0.84

P-values show results of ANOVA for comparison between ErrP and CorrP within control and SCI group, respectively, and between control and SCI group for ErrP and CorrP, respectively (from left to right).‘*' indicates significance of differences.

Overall morphology of the ErrP was comparable between groups, as ErrPs consisted of three characteristic peaks in subjects with and without SCI (Figure 5). However, individuals with SCI showed significant smaller mean amplitudes for wN1 and wP2 at site FCz and Cz compared to control subjects (Figure 5, Table 2). The CorrPs were not significantly different between groups at FCz or Cz for wP1, wN1 or wP2 (Figure 5, Table 2). Repeated-measurement ANOVA showed no significant difference between runs in each group (“repetition” ^*^ “group”) and between runs with and without influence of subjects on the feedback (“influence” ^*^ “group”). Mean voltage values of wP1, wN1 and wP2 did not correlate significantly with either chronicity after SCI or the level of injury.

A comparison of the amplitudes between the subgroup of SCI individuals (*n* = 2) who did not receive CNS active medication with the subgroup (*n* = 9) who received CNS agents revealed no significant differences.

## Discussion

To our knowledge, this is the first study systematically investigating the electrophysiological correlates in the EEG of error recognition in subjects with SCI and in age- and sex-matched able-bodied controls.

There was no significant difference of the mean amplitude of the ErrP between conditions where study participants were able to influence the feedback (condition “influence”) and conditions where the system displayed a certain amount of errors independently from the joystick input. This finding suggests that our approach of fully controlling error counts is a legitimate experimental condition and implies that the higher error count in the “influence” condition has no impact on the ErrP. This is corroborated by Falkenstein et al., who did not find a correlation between Ne/ERN (error negativity/error-related negativity, a negative potential elicited by errors similar to the “response ErrP”) amplitude and error rate (39). In view of these results, it is unlikely that the lower amplitude of the ErrP in subjects with SCI compared to the able-bodied controls under the experimental condition “influence” might have been caused by the (non-significant) higher number of errors.

### Effects of SCI on Morphology of the ErrP

The experimental setup based on visual feedback about the correctness of the direction of visual cue-based shoulder movements proved to evoke ErrPs in the EEG of able-bodied individuals with characteristic peaks described previously (16, 37).

P1 occurred early after feedback presentation and coincided with the potential measured in the VF run during which only visual stimuli without a preceding task were presented (Figures 2, 3). Since there was no significant difference of P1 between the error and the correct condition, it is likely that P1 represents neural activity associated to general stimulus processing and is not related to error processing.

The ErrP was diminished in amplitude in subjects with SCI. Accordingly, the difference between ErrP and CorrP was significant in the control but not in the SCI group. Since we controlled for participants' age and sex, this alteration is most likely associated to and with the consequences of the SCI.

Our results suggest that changes in the electrophysiological markers of error processing are caused by the deafferentation/deefferentation of the brain by the SCI and/or its consequences on CNS reorganization. However, little is known about the onset and course of these changes after SCI. Likewise, it is unknown whether changes in the ErrP represent an unspecific reaction to deafferentation and deefferentation of the brain due to SCI or if these changes are a result of ongoing CNS and in particular brain plasticity (46). It needs to be shown in future longitudinal studies assessing patients a few weeks after the injury until the chronic phase up to 1 year, to which extent the course of the decrease of ErrP amplitudes might serve as a marker for brain reorganization. We could not find a significant correlation between ErrP amplitude and level or chronicity of SCI, but our study cohort was likely too small to detect any influence of these factors. Additionally, the majority of our participants with SCI had a motor complete injury (Table 1). Therefore, the influence of the severity and level of the lesion as well as the time after onset of SCI on ErrPs remains unclear. While our study results from humans show that SCI has a general impact on ErrPs, longitudinal studies investigating the course of ErrPs after acute SCI need to be conducted in the future to gain further insights into the underlying neurophysiological mechanisms of this SCI–induced change of ErrPs.

### Potential Confounding Factors Influencing ErrPs

Since individuals with SCI show a high risk to develop spasticity (47) or chronic pain (48), they often take many medications such as spasmolytic drugs, anticonvulsants, and pain medication including antidepressants. It has been shown that spasmolytic medications such as anticholinergics for treatment of an overactive detrusor muscle, in particular Oxybutinin, and other medication for treatment of spasticity of skeletal muscles such as baclofen, an agonist to GABA-β receptors, affect CNS activity and thus have an influence on the EEG spectral power distribution. This includes a decrease in alpha, beta, and theta activity (49–52) and an increase in delta activity (53). Pregabalin is an anticonvulsant binding to a subunit of the voltage-gated calcium channels in CNS tissues and by this reducing calcium influx at nerve terminals, which may inhibit the release of excitatory neurotransmitters (54). Individuals with chronic pain and intake of Pregabalin show increase in theta and delta activity (55). Opioids produce analgesic effects on neurons by directly acting on receptors located on neuronal cell membranes (56). The intake of opioids causes an increase in delta activity and decrease in theta and alpha activity in healthy subjects (57). An increase in theta as well as delta activity is known to be associated with an increased level of mental fatigue (58).

In our study, a comparison of the ErrPs of individuals with SCI and CNS active medication to those without did not show any differences. This indicates that the decrease of ErrPs amplitudes in the SCI group is rather a consequence of the SCI than of the CNS agents. However, due to the small number of study participants and the unbalanced distribution of medication within the SCI group, this statement should be interpreted very carefully and needs to be confirmed in a larger study involving more study participants with SCI. Under any circumstances, such an investigation will be most challenging, because most individuals with SCI receive CNS activity-modulating medications. In general, there is an urgent need to further investigate the relationship of different types and combinations of medications on EEG potentials. Similar to our study, other studies found no correlation between medication and changes of the Ne/ERN (59–61).

It should be noted, that changes in the ErrP have been shown for different pathological conditions. ErrPs are diminished in patients affected by brain injuries like lesions of the prefrontal cortex (62). Additionally, it has been found that EEG parameter related to error processing are altered in psychiatric diseases: while schizophrenia has been shown to be accompanied with diminished Ne/ERN (63–65), patients with obsessive compulsive disorders or depression had increased Ne/ERN (59, 66). Effect of medication was discussed in all these studies, but no final conclusion was made.

### Implications for Use of ErrPs in Hybrid-BCIs

The main goal of our study has been to investigate whether there are any differences in the morphology of ErrPs between people with SCI and sex- and age-matched able-bodied subjects. Due to our limited technical possibilities, we were not able to implement a single trial ErrP detection algorithm in our experimental setup to perform a second experiment with online, single trial error correction. Therefore, our study lacks the quantitative comparison of this online correction on the performance of the SCI and the control group.

However, in general, only a few groups investigated the impact of an online error correction on BCI-performance. Additionally, only very limited data is available from experiments involving end users with severe motor impairments. Margaux et al. were able to show in able-bodied subjects that automatic error correction in a P300 speller yielded a higher bit rate than a respelling strategy. Interestingly, their experiments clearly distinguished two groups who differed according to individual specificity in ErrP detection. The high specificity group had larger evoked responses and made fewer errors which were corrected more efficiently (67). In the light of these results, the worse signal-to-noise ratio of the ErrPs in our SCI group compared to the control group will most probably result in a less effective error correction. This assumption is confirmed by the results of a recent paper proposing a P300-based BCI speller which combined a double ErrP detection to automatically correct erroneous decisions (24). The tests of this speller with 9 able-bodied subjects and 1 individual with tetraplegia due to a cervical SCI showed that the post-correction accuracy was lowest in the individual with SCI. Unfortunately, the authors reported neither on the time after SCI of this person nor on alterations of the ErrP. However, based on our results it can be assumed that the lower amplitudes of this individual's ErrPs were the reason for the lower online correction accuracy.

In another study with a P300-speller involving 5 individuals with Amyotrophic Lateral Sclerosis (ALS) and one with Duchenne muscular dystrophy, the ErrP classification increased performance with only slightly worse results of the group with motor impairments compared to the group of 17 able-bodied subjects (68). Interestingly, in this study there were no significant differences between the groups for peak latency or peak-to-peak amplitudes. This observation underlines the substantial influence of an altered ErrP morphology on the classification accuracy.

Nevertheless, further investigations are needed to evaluate whether ErrPs can be successfully included in an online control scheme and whether such algorithms achieve a level of matureness to be transferred in the everyday life of end users with SCI (69, 70).

### Other Implications of the Obtained Results

We were primarily interested in ErrPs of people with SCI for their implementation in hBCIs, so we did not monitor whether the changes in ErrP amplitudes were accompanied by changes of general cognitive functions. There has been a remarkable interest about EEG-based correlates of error processing in neuroscientific and psychological studies long before it has been investigated in BCI-research (14, 71). During review of these older studies, it becomes obvious that a different nomenclature is used for the same mechanisms in different fields of research: The terms Ne/ERN (error negativity/error-related negativity) and Pe (error positivity), for example, are used to describe the two peaks of a potential which occurs when for instance a choice reaction time task is conducted. In this type of task, the subject has to quickly decide, after the presentation of a stimulus, for the correct one out of several options (39). These potentials may best be described as “response ErrP” in the BCI field (17). The Ne/ERN has a different morphology than the ErrP investigated in this study. Nevertheless, it is still likely that our findings have implications in the context of the Ne/ERN-research as many different EEG manifestations of error processing share localization in the medial frontal cortex (72). Additionally, they show similar time-frequency characteristics like bursts of activity in the 4–7 Hz EEG frequency range, although there are some topographical differences (73). Although the clinical significance of the Ne/ERN remains unclear [e.g., (74)], it is widely accepted that the Ne/ERN is the EEG correlate of the activity of a neural network that monitors action and subsequently induces error corrections (74). Studies involving individuals with psychological disorders and an associated overactive error monitoring have shown a positive correlation between an increased feeling of responsibility and the ERN amplitude (75). Transferring this knowledge to our study, it consequently might be hypothesized that reduced ErrP in persons with SCI is associated with a generally worse action monitoring and higher error rate compared to able-bodied individuals. This generally lower cognitive capacity has been confirmed in a recent study assessing individuals with SCI from the early subacute to the chronic stage (76). Ultimately, the ErrP, respectively the Ne/ERN, may qualify as a (predictive) marker for the risk and the course of developing deficits for abilities such as attention span, initial learning, concentration, memory function or problem solving (77). However, this also needs to be confirmed in future clinical trials.

## Conclusion

In conclusion, our study revealed that the morphology of ErrPs was comparable in individuals with and without SCI. However, mean amplitudes of time windows around N1 and P2 were reduced in the SCI group compared to sex- and age-matched able-bodied controls. Future longitudinal studies need to clarify if this is a direct consequence of the deafferentation/defferentation associated to the SCI or a result of neural plasticity after SCI. It has to be further determined, whether this phenomenon has a negative impact on the use of ErrPs in brain-computer interfaces for real-time control of assistive devices.

## Author Contributions

PK developed the study design, assisted in the hardware development, in providing the documents for ethical approval, performed the experimental work, and data analysis. MS developed software and hardware for the study and gave support for the study design. CS gave major support to the statistical analysis. SF obtained informed consent from the study participants with SCI and provided the patients' history and medication. MH supported the experimental work and gave input and advice for data analysis. NW and RR supervised the complete design of the project, provided major help in analyzing the data, contributed to ethics approval, and trial registration. All authors contributed to the drafting and revision of the manuscript.

### Conflict of Interest Statement

The authors declare that the research was conducted in the absence of any commercial or financial relationships that could be construed as a potential conflict of interest.
